# The neurophysiology of biological motion perception in schizophrenia

**DOI:** 10.1002/brb3.303

**Published:** 2014-12-18

**Authors:** Carol Jahshan, Jonathan K Wynn, Kristopher I Mathis, Michael F Green

**Affiliations:** 1 Mental Illness Research, Education and Clinical Center (MIRECC), VA Greater Los Angeles Healthcare SystemLos Angeles, California; 2 Semel Institute for Neuroscience and Human Behavior, University of CaliforniaLos Angeles, California

**Keywords:** Biological motion, electroencephalography, event-related potential, schizophrenia, visual perception

## Abstract

**Introduction:**

The ability to recognize human biological motion is a fundamental aspect of social cognition that is impaired in people with schizophrenia. However, little is known about the neural substrates of impaired biological motion perception in schizophrenia. In the current study, we assessed event-related potentials (ERPs) to human and nonhuman movement in schizophrenia.

**Methods:**

Twenty-four subjects with schizophrenia and 18 healthy controls completed a biological motion task while their electroencephalography (EEG) was simultaneously recorded. Subjects watched clips of point-light animations containing 100%, 85%, or 70% biological motion, and were asked to decide whether the clip resembled human or nonhuman movement. Three ERPs were examined: P1, N1, and the late positive potential (LPP).

**Results:**

Behaviorally, schizophrenia subjects identified significantly fewer stimuli as human movement compared to healthy controls in the 100% and 85% conditions. At the neural level, P1 was reduced in the schizophrenia group but did not differ among conditions in either group. There were no group differences in N1 but both groups had the largest N1 in the 70% condition. There was a condition × group interaction for the LPP: Healthy controls had a larger LPP to 100% versus 85% and 70% biological motion; there was no difference among conditions in schizophrenia subjects.

**Conclusions:**

Consistent with previous findings, schizophrenia subjects were impaired in their ability to recognize biological motion. The EEG results showed that biological motion did not influence the earliest stage of visual processing (P1). Although schizophrenia subjects showed the same pattern of N1 results relative to healthy controls, they were impaired at a later stage (LPP), reflecting a dysfunction in the identification of human form in biological versus nonbiological motion stimuli.

## Introduction

Social cognition, the mental operations that underlie social interactions, is impaired in schizophrenia (Penn et al. 2006). This impairment has been linked to diminished social functioning and spans several cognitive domains, including perceiving, interpreting, and generating responses to socially relevant stimuli (Couture et al. 2006). One fundamental aspect of social cognition is recognizing that a situation actually involves other people. In natural scenes, this recognition is accomplished in part through the ability to detect characteristic human form and movement within a visual context, i.e. human biological motion (Johansson 1973). Individuals with schizophrenia have deficits in the ability to recognize and detect human biological motion (Kim et al. 2011, 2013; Spencer et al. 2013). More specifically, when briefly presented with point-light animations of a human figure engaged in a familiar activity or scrambled point-lights (in which the global percept of a human form has been disrupted), individuals with schizophrenia have trouble discriminating biological motion from nonbiological motion. This deficit has been shown to be associated with poor social functioning (Kim et al. 2005). However, little is known about the neural underpinnings of dysfunctional biological motion in schizophrenia.

In healthy individuals, the perception of human biological motion is rapid and effortless. Many cortical regions, identified using functional imaging (fMRI), have been implicated in the processing of human biological motion including, but not limited to, the posterior superior temporal sulcus (STSp; Grossman et al. 2000), fusiform gyrus (Grossman and Blake 2002), amygdala (Bonda et al. 1996), and mirror neuron networks in the premotor cortex (Saygin et al. 2004). The precise timing of information processing across this cortical network can be assessed using electroencephalography (EEG), given its high temporal resolution. EEG studies that have been conducted in healthy individuals point to specific phases of activation during the viewing of human biological motion versus random or nonhuman motion. Distinct stages of biological motion processing as assessed with event-related potential (ERP) components include: P1, N1, and the late positive potential (LPP).

Processing of biological motion can start as early as the P1 component, which peaks around 100 msec after stimulus onset and is maximal at occipital electrodes, with a greater amplitude to biological compared to nonbiological motion stimuli (Hirai et al. 2009; Krakowski et al. 2011). This early processing stage seems to be associated with bottom-up stimulus feature processing (Buzzell et al. 2013) and is not always specific to the perception of human motion (Kroger et al. 2014). Following P1, a negative-going deflection, the N1 (sometimes referred to as N170 or N200), peaks at approximately 200 msec at occipito-temporal sites (Hirai et al. 2003, 2005; Jokisch et al. 2005). N1 is usually stronger to biological compared to nonbiological motion stimuli and is thought to reflect the integration of form and motion processing (Baccus et al. 2009). However, results from a recently published study (White et al. 2014) suggest that the N1 is not specific to biological motion perception but rather reflects processing of objects in general. A later ERP component that is sensitive to both human form and motion information, the medial posterior positivity/ventral-lateral negativity (MPP/VAN), has also been identified (White et al. 2014). The MPP is similar to the slow LPP wave (also referred to as P400+) that has been found in previous studies and shown to be enhanced during human motion processing. The LPP is seen between approximately 400–700 msec and is maximal at centro-parietal electrodes (Krakowski et al. 2011; Kroger et al. 2014). This last ERP component reflects top-down cognitive processing or active decoding of stimulus content (Krakowski et al. 2011). It has also been related to sustained attentional processing of motivationally relevant stimuli (Hajcak et al. 2010). Furthermore, this component has been suggested to have a generator in STSp (White et al. 2014).

To date there have been no ERP studies of biological motion in schizophrenia. Therefore, we do not know if one or all stages of biological motion processing are dysfunctional in this clinical population. An fMRI study of biological motion in schizophrenia (Kim et al. 2011) found that individuals with schizophrenia had comparable levels of event-related activations in STSp to biological and scrambled motion stimuli, while healthy subjects exhibited stronger STSp activation to biological motion only. The authors argued that subjects with schizophrenia may overprocess randomly moving dots or see meaning when there is none, which can have negative social consequences.

The aim of the present study was to conduct a temporal assessment of the neural mechanisms underlying the detection of human movement in schizophrenia. Given previous findings that P1 and N1 are generally reduced in schizophrenia and are not sensitive to biological motion perception, we hypothesized we would find overall group differences, but no group by condition interaction for these waveforms. Based on behavioral and fMRI findings of biological motion in schizophrenia, we hypothesized that LPP amplitude would be larger to biological motion compared to nonbiological motion in controls, while people with schizophrenia will show no differential effects of biological motion on this ERP component.

## Methods

### Participants

Twenty-four individuals with schizophrenia and 18 healthy control subjects participated in the study. All subjects meeting the following criteria were eligible for participation: between the ages of 18 and 60, IQ over 70 based on chart review, normal or corrected-to-normal vision, and sufficiently fluent in English to understand the procedures. Subjects were excluded if they had substance dependence in the last 6 months or substance abuse in the last month, history of head injury (with loss of consciousness for >15 min) or an identified neurological condition. All subjects provided written informed consent after study procedures were fully explained in accordance with procedures approved by the Institutional Review Board at the Veterans Affairs Greater Los Angeles Healthcare System (VAGLAHS).

Individuals with schizophrenia were recruited from outpatient treatment clinics at the VAGLAHS and from board-and-care residences in the community. Diagnosis was based on the Structured Clinical Interview for DSM-IV Axis I Disorders (SCID-I; First et al. 1997). Psychiatric symptoms were evaluated using the expanded 24-item UCLA version of the Brief Psychiatric Rating Scale (BPRS; Ventura et al. 1993). For the BPRS, we report the total score and means for the “positive symptom” and “depression/anxiety” factors (Kopelowicz et al. 2008). All the clinical assessments were conducted by interviewers trained to reliability by the Treatment Unit of the VISN 22 Mental Illness Research, Education, and Clinical Center (MIRECC) based on established procedures (Ventura et al. 1993, 1998). Schizophrenia subjects were considered to be clinically stable, defined as no psychiatric medication changes in the past 6 weeks, no inpatient hospitalization in the past 3 months, and no changes in housing in the past 2 months. Twenty of these individuals were receiving atypical antipsychotic medications, two were receiving typical antipsychotic medications, one was receiving both types of antipsychotics, and one was not taking antipsychotic medication at the time of assessment.

Healthy controls were recruited through Internet advertisements and were screened with the SCID-I and SCID-II (First et al. 1996). They were excluded if they met criteria for any lifetime psychotic disorder, current Axis I mood disorder, recurrent depression, avoidant, schizoid, schizotypal, or paranoid personality disorder, or if they reported a history of psychosis in a first-degree relative.

### Procedures

Participants completed a human biological motion task (Kim et al. 2011) while their EEG was simultaneously recorded. Stimuli consisted of 12 black dots presented on a white background at central fixation presented on an LCD monitor at 75 Hz situated 1 m from the subject. A fixation cross was continuously present throughout the experiment. The stimulus clip was then presented for 1 sec. After stimulus offset, there was a 1 sec delay before the screen prompted the subject to make their response. Subjects were asked to decide whether the clip resembled human or nonhuman movement by pressing a corresponding button. Subjects had unlimited time to make their response. After the response, there was a 0.5 sec delay before the next trial began.

**Figure 1:**
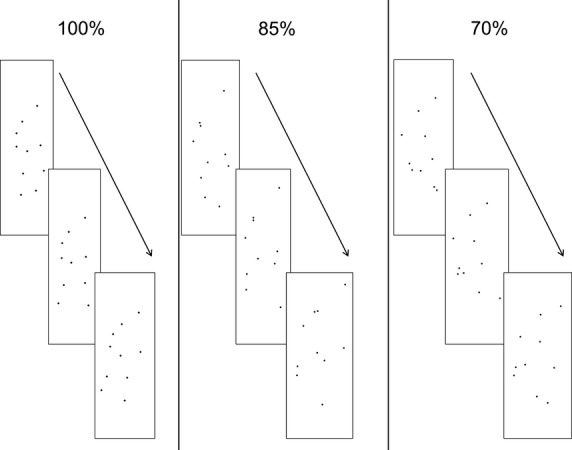
Examples of point-light animations depicting 100%, 85%, and 70% biological motion. Three consecutive frames are shown for each condition.

The dots were arranged and animated in a manner that corresponded to human (e.g., walking, jumping) or nonhuman movement (see Fig.1). The difficulty level was manipulated by scrambling the movement of the animations. Briefly, stimuli were scrambled by resetting the position of the dots in the first frame somewhere between the original and a completely randomized location, while maintaining the original motion trajectory of each dot. Refer to Kim et al. (2011) for a more complete description of how random motion was introduced into the stimuli. The three levels of difficulty were 100% biological motion, 85% biological motion, and 70% biological motion. Two blocks of trials (a practice block followed by a test block) were presented twice. In the practice block, 10 trials of 100% and 10 of 70% were shown to familiarize the participants with the task. The test block consisted of 40 trials for each type of trial (100%, 85%, 70%), for a total of 120 trials per block (240 total trials for session). The proportion of trials classified as human movement by level of difficulty was the primary dependent behavioral measure.

### EEG recording and analysis

Electroencephalography (EEG) recordings were acquired with a 64-channel BioSemi ActiveTwo amplifier (Biosemi B. V., Amsterdam, the Netherlands). Additional electrodes were placed above and below the left eye and at the outer canthi of both eyes to monitor blinks and eye movements. Each active electrode was measured online with respect to a common mode sense electrode during data collection, forming a monopolar channel. Data were sampled at 1024 Hz with a bandpass of 0–100 Hz and were re-referenced offline to the averaged mastoid reference.

Event-related potential data processing was performed using BrainVision Analyzer 2 (Brain Products, Gilching, Germany). A high-pass filter at 0.1 Hz (zero phase shift, 12 dB/octave rolloff) was applied to the raw data. Based on visual inspection, bad electrodes were removed from the recording and a spherical spline interpolation was used to recreate the electrode (Perrin et al. 1989; Picton et al. 2000). Eyeblinks were removed from the data using a regression-based algorithm (Gratton et al. 1983). Data were low-pass filtered at 30 Hz (zero phase shift, 24 dB/octave rolloff), epoched at −100 to 700 msec relative to stimulus onset, and baseline corrected to the average of the prestimulus interval. Epochs that contained activity exceeding ±75 *μ*V at any electrode were automatically rejected. All trials (collapsed across those identified either as human or nonhuman) were included in the analysis. The mean (SD) number of accepted trials was 83% (11.7) for controls and 83% (14.8) for schizophrenia subjects.

Electrodes and time windows were defined based on our review of prior studies of biological motion (Krakowski et al. 2011; White et al. 2014) as well as visual inspection of our topographical maps. The first positive peak (P1) was measured as the mean activity at P5, P7, PO7 (left hemisphere) and P6, P8, PO8 (right hemisphere) in the 130–150 msec latency range. The first negative peak (N1) was measured between 190 to 210 msec at P7, PO7, O1 (left hemisphere) and P8, PO8, O2 (right hemisphere). The LPP was measured as the mean activity between 400 to 700 msec at pooled centro-parietal electrodes (Cz, CPz, Pz, C1, C2, CP1, CP2, P1, P2).

### Statistical analyses

Independent samples *t*-tests and chi-square tests were used to assess group differences for continuous and categorical demographic variables, respectively. For the behavioral data we conducted a 3 × 2 repeated measures analysis of variance (rmANOVA) with condition as the within-subject factor and group as the between-subject factor. For the ERP data, two separate rmANOVAs with condition and hemisphere as within-subject factors and group as a between-subject factor were conducted to assess group differences separately in P1 and N1. A rmANOVA with condition and group as factors was performed for LPP. Greenhouse-Geisser corrections (*ε*) were used in the rmANOVAs that contained more than one degree of freedom to correct for nonsphericity. We report the uncorrected degrees of freedom, the corrected *P*-value, and the correction factor *ε*. Follow-up Bonferonni-corrected *t*-tests were used to examine significant main effects or interactions. Relationships between the ERPs and behavioral performance were investigated using Pearson correlations within each group. An alpha level of *P* = 0.05 was used for all analyses.

## Results

### Demographic and clinical characteristics

Demographic and symptom ratings can be seen in Table 1. The groups were matched on age, gender distribution, and race. Individuals with schizophrenia had significantly fewer years of education than controls, *t*(40) = 3.79, *P* = 0.001. Although the groups did not significantly differ on parental education (*P* = 0.06), schizophrenia subjects had fewer years of parental education than controls. They also had relatively mild levels of symptoms.

**Table 1:** Demographic and clinical characteristics

	Healthy controls (*N* = 18)	Schizophrenia subjects (*N* = 24)
Age (Mean/SD)	45.2 (6.9)	46.9 (10.7)
Gender (% male)	72	79
Personal education (Mean/SD)**	14.9 (1.3)	13.0 (1.8)
Parental education (Mean/SD)	13.9 (2.6)	11.7 (3.9)
BPRS total (Mean/SD)	–	35.4 (9.1)
BPRS positive symptom (Mean/SD)	–	11.4 (5.2)
BPRS depression/anxiety (Mean/SD)	–	6.3 (2.6)

BPRS, Expanded Brief Psychiatric Rating Scale;

** *P* < 0.001.

### Behavioral performance

The dependent variable for this analysis was the percentage of trials identified as biological motion. The ANOVA revealed a significant main effect of condition, *F*(2, 80) = 547.03, *P* < 0.001, *ε *= 0.88, and a significant condition × group interaction, *F*(2, 80) = 5.62, *P* = 0.007, *ε *= 0.88. There was no significant main effect of group. Performance was significantly different among all three conditions (all *P*'s < 0.001), with performance best at 100% and worse at 70%, with 85% in between. The interaction was due to schizophrenia subjects identifying significantly fewer trials as human movement than controls in the 100% and 85% conditions (*P*'s < 0.05) but not in the 70% condition, in which both groups performed comparably (see Table 2).

**Table 2:** Group means by condition for behavioral performance and event-related potentials

	100%	85%	70%
Healthy controls (*N* = 18)			
Behavior**	86% (8%)	61% (17%)	8% (10%)
P1	2.33 (2.21)	2.49 (2.51)	2.33 (2.36)
N1	−2.63 (3.04)	−2.38 (2.87)	−3.81 (3.42)
LPP*	4.87 (3.97)	3.71 (3.55)	2.95 (2.92)
Schizophrenia subjects (*N* = 24)			
Behavior**	78% (13%)	50% (16%)	11% (14%)
P1	0.66 (2.21)	0.98 (2.51)	1.20 (2.36)
N1	−1.54 (3.05)	−1.29 (2.87)	−1.83 (3.42)
LPP	4.67 (3.97)	4.39 (3.80)	4.43 (3.57)

* LPP, late positive potential; Significant differences at *P *<* *0.05

(*) *P *<* *0.001 (**) among conditions; Behavior = percent of trials identified as biological motion.

We also conducted a d-prime analysis of the behavioral data as a measure of separation between conditions (rather than as a measure of accuracy), comparing the 100% and 85% conditions each to the 70% condition. The schizophrenia group had significantly smaller d-prime values relative to the control group for the 100% condition, 2.26 (0.71) versus 2.71 (0.44), *t*(40) = 2.38, *P* = 0.02 and the 85% condition, 1.43 (0.47) versus 1.89 (0.40), *t*(40) = 3.34, *P* = 0.002.

### ERP results

**Figure 2:**
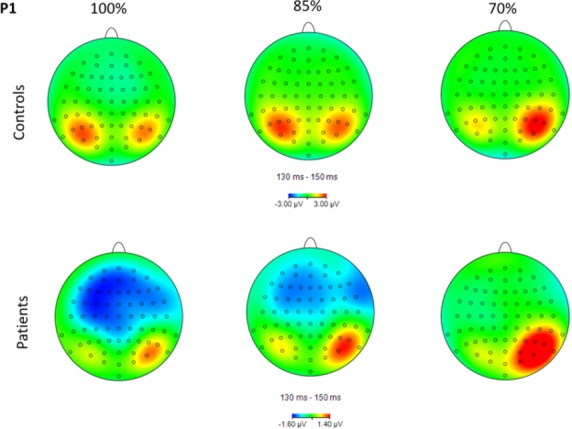
Topographical maps of P1 activity in the 130–150 msec range for each group and condition. Note that the scale is different for the two groups.

Topographical maps of P1, N1, and LPP activity for each group and condition are shown in Figures2─4, respectively. Group means by condition for each ERP component are shown in Table 2. For the P1, there was a significant main effect of group, *F*(1, 40) = 4.24, *P* = 0.046, main effect of hemisphere, *F*(1, 80) = 7.26, *P* = 0.01, and condition × hemisphere interaction, *F*(2, 80) = 20.35, *P* < 0.001, *ε *= 0.97. Controls had a significantly larger P1 compared to schizophrenia subjects, 2.39 (2.56) *μ*V versus 0.95 (1.97) *μ*V, respectively. P1 was significantly larger in the right compared to left hemisphere, 2.04 (2.44) *μ*V versus 1.30 (2.54) *μ*V, respectively. The interaction was due to P1 being significantly larger in the right compared to the left hemisphere for the 70% condition only (*P* < 0.001).

**Figure 3:**
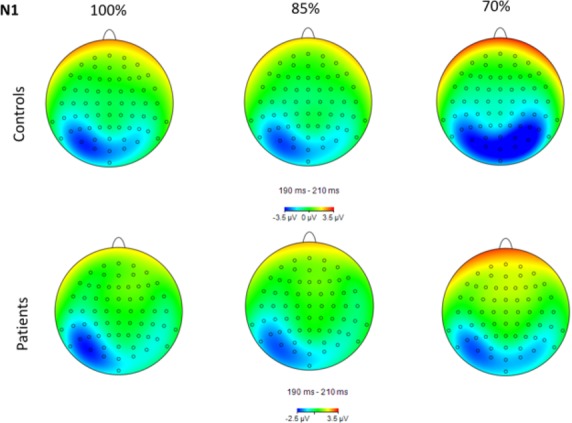
Topographical maps of N1 activity in the 190–210 msec range for each group and condition. Note that the scale is different for the two groups.

**Figure 4:**
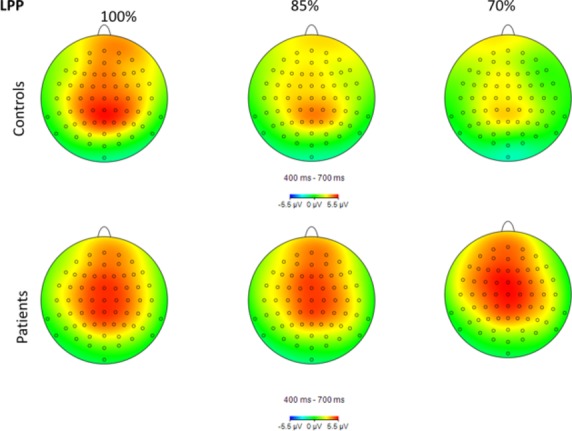
Topographical maps of late positive potential (LPP) activity in the 400–700 msec range for each group and condition.

**Figure 5:**
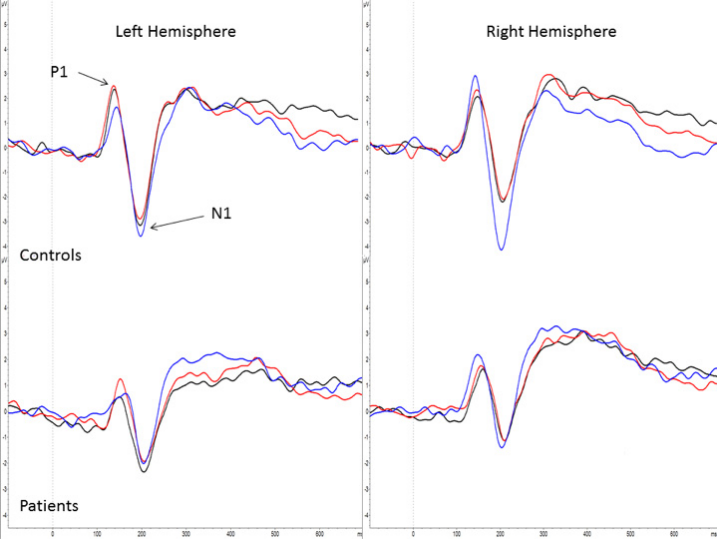
Grand average P1/N1 event-related potentials in response to 100% (black line), 85% (red line), and 70% (blue line) biological motion for controls (upper panel) and schizophrenia individuals (lower panel). The waveforms are shown at pooled electrodes P6, P8, PO8, and O2 over the right hemisphere and P5, P7, PO7, and O1 over the left hemisphere.

For the N1, there was a significant main effect of condition, *F*(2, 80) = 7.88, *P* = 0.001, *ε *= 0.96, main effect of hemisphere, *F*(1, 80) = 4.97, *P* = 0.03, and condition × hemisphere interaction, *F*(2, 80) = 26.11, *P* < 0.001, *ε *= 0.86. The main effect of group was not significant. N1 was significantly larger in the 70% condition (−2.82 (3.45) *μ*V) compared to the 100% (−2.09 (3.08) *μ*V, *P* < 0.05) and 85% conditions (−1.84 (2.90) *μ*V, *P* < 0.001). N1 was significantly larger in the left compared to the right hemisphere, −2.64 (3.58) *μ*V versus −1.86 (2.78) *μ*V, respectively. The interaction was due to N1 being significantly larger in the left compared to the right hemisphere for the 100% (*P* = 0.001) and 85% (*P* = 0.005) conditions only. Figure5 shows grand average P1/N1 waveforms for each group and condition.

**Figure 6:**
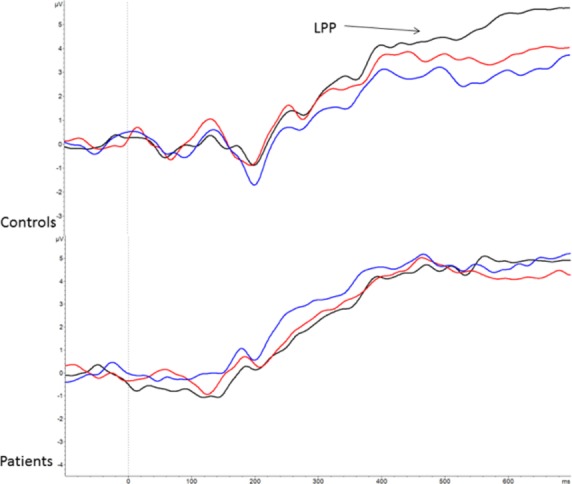
Grand average late positive potential (LPP) waveforms at pooled electrodes Cz, CPz, Pz, C1, C2, CP1, CP2, P1, P2 for controls (upper panel) and schizophrenia individuals (lower panel). Black indicates 100% biological motion. Red and blue indicate 85% and 70% biological motion, respectively.

For the LPP, there was a significant main effect of condition, *F*(2, 80) = 6.99, *P* = 0.002, *ε *= 0.92, and condition × group interaction, *F*(2, 80) = 4.09, *P* = 0.02, *ε *= 0.92. The LPP was larger in the 100% condition (4.77 (4.01) *μ*V) compared to the 85% (4.05 (3.73) *μ*V, *P* < 0.05) and 70% conditions (3.69 (3.35) *μ*V, *P* < 0.01). The interaction was due to controls having a significantly larger response in the 100% condition compared to the 85% (*P* = 0.01) and 70% conditions (*P* = 0.001); there were no significant differences among conditions in the schizophrenia group. Figure6 shows grand average LPP waveforms for each group and condition.

### Correlations between ERPs and behavioral performance

There were significant correlations in both groups between behavioral performance and LPP, but not P1 or N1. Within the patient group, in the 100% condition better performance correlated with a larger LPP response (*r* = 0.43, *P* = 0.04). Within the control group, in the 70% condition poorer performance correlated with a larger LPP response (*r* = −0.59, *P* = 0.01).

## Discussion

This is the first study to our knowledge to examine the time course of biological motion processing in individuals with schizophrenia compared to healthy controls. At the behavioral level, schizophrenia subjects identified fewer trials as human movement than controls in the 100% biological motion condition, but performed comparably in the 70% condition. At the neural level, we found significant group differences for the P1, reflecting a deficit at the earliest stage of visual processing. However, P1 amplitude reduction in schizophrenia subjects was not sensitive to biological motion perception, as there were no differential effects in the coherent biological motion condition versus the noncoherent conditions. At the second stage, there were no group differences in N1 amplitude but there was a condition main effect: Both groups had the largest N1 in the 70% condition. The most striking group differences were observed at the latest stage of processing over the centro-parietal region. While controls showed a larger LPP amplitude in the coherent biological motion condition relative to the noncoherent conditions, the schizophrenia group' LPP was not modulated by coherent versus noncoherent biological motion stimuli. Moreover, the ability of schizophrenia individuals to correctly identify coherent biological motion as human movement was correlated with the magnitude of their LPP response, but not with the earlier components. However, this correlation should be interpreted with caution given that it would not survive correction for multiple tests.

Our pattern of results suggests that the processing of biological motion begins in the latency range of the N1 component which peaks approximately 200 msec following stimulus onset. Our healthy control sample did not show a difference in P1 when processing coherent versus noncoherent biological motion stimuli, which is consistent with one prior study (Kroger et al. 2014) but inconsistent with two others (Krakowski et al. 2011; Buzzell et al. 2013). The larger N1 effect in the 70% condition across groups went in the opposite direction to some previous studies (Hirai et al. 2003, 2009; Jokisch et al. 2005) that showed greater negativity in response to biological than nonbiological motion stimuli. However, these studies employed paradigms that differed from ours in terms of stimulus characteristics and task demands (e.g., passive viewing or identification of a static display). Our N1 finding is similar to a more recent study (White et al. 2014) that showed larger N1 to scrambled stimuli than to upright human forms in healthy subjects. In fact, N1 has been shown to be larger during more visually demanding tasks (Luck et al. 1990; Hillyard and Anllo-Vento 1998), which may explain the N1 finding in the current study: Amplification of N1 in the 70% or most difficult condition may reflect the need to allocate more attentional effort to determine whether the stimuli moving noncoherently resembled human movement.

As for the LPP, which is thought to index human action recognition and more elaborate processing of biological motion (Hajcak et al. 2010), the data do not show a differential LPP response between biological and nonbiological motion stimuli in people with schizophrenia. This finding suggests that a disturbance in the recognition of particular human actions and/or their meaning may underlie the schizophrenia subjects' poor behavioral performance. Although the schizophrenia subjects' LPP response was similar in magnitude to that of controls in the 100% condition, it did not decrease with less salient biological information, resulting in greater amplitudes than controls in the 85% and 70% conditions.

The controls' higher LPP amplitude in the 100% condition relative to the other conditions is consistent with the LPP reflecting processes of action recognition. Schizophrenia individuals, however, seem to be processing all stimuli the same. Interestingly, a similar pattern of results using fMRI has been observed in schizophrenia individuals in the STSp (Kim et al. 2011), a brain region known to be involved in the perception of biological motion and registration of socially relevant sensory information (Grossman et al. 2000). These subjects had a strong, undifferentiated STSp activation to both biological and scrambled motion. The authors argued that individuals with schizophrenia tend to identify biological motion in stimuli where it is not actually present, which may lead them to misinterpret the actions of others. The overall high LPP amplitudes in people with schizophrenia in the current study, combined with similar findings previously reported in the STSp, suggest that high levels of LPP activity may be triggered by stimuli possibly containing biological motion, but schizophrenia individuals are poor at accurately sorting biological from nonbiological patterns, leading to higher error rates.

The study has a few limitations. First, our groups were not matched on personal or parental education. However, these variables were largely uncorrelated with our behavioral and ERP measures, except for one significant association between parental education and P1 in the healthy control group. Second, our sample consisted of older chronic schizophrenia subjects who were receiving antipsychotic medications at the time of testing, which raises the question of whether our results generalize to a younger, recent-onset, or unmedicated sample. Third, one limitation of our biological motion task is that there is no right or wrong answer in the 85% and 70% conditions. Accuracy can only be determined in the 100% condition, which had a very small number of incorrect trials for both groups. For this reason, we included all trials in the ERP analysis whether they were classified as human or nonhuman. Finally, we were unable to find a consistent use of any specific reference electrode in the biological motion literature. ERP studies of biological motion have used different references, including average (Hirai et al. 2003, 2005; Kroger et al. 2014), nose (Jokisch et al. 2005; Hirai et al. 2009), Cz (White et al. 2014) and FPz (Krakowski et al. 2011). In the current study, we used the mastoids as reference because it was the approach we have used previously with schizophrenia subjects.

In summary, our results suggest that people with schizophrenia do not perceive biological motion as well as healthy controls. The impairment does not seem to be explained by a disruption at the initial neural stages of biological motion processing (P1 and N1). It is at a later, higher level of processing (LPP) that individuals with schizophrenia fail to modulate their neurophysiological response, which suggests a dysfunction in the recognition of human form in biological motion versus nonbiological motion stimuli.
